# Modeling esophageal protection from radiofrequency ablation via a cooling device: an analysis of the effects of ablation power and heart wall dimensions

**DOI:** 10.1186/s12938-020-00821-z

**Published:** 2020-10-12

**Authors:** Marcela Mercado, Lisa Leung, Mark Gallagher, Shailee Shah, Erik Kulstad

**Affiliations:** 1 Bioengineering Department, Engineering Faculty, Universidad de Antioquia UdeA, Calle 70 No. 52-21, Medellin, Colombia; 2 St. George’s University Hospitals NHS Foundation Trust, St. George’s, University of London, Cranmer Terrace, Tooting, London, SW17 0RE UK; 3 Illinois Institute of Technology, Chicago, USA; 4 Southwestern Medical Center, University of Texas, Dallas, TX USA

**Keywords:** Atrial fibrillation, Radiofrequency ablation, Esophageal protection, Finite element model, Mathematical modeling, Esophageal cooling, Ablation parameters

## Abstract

**Background:**

Esophageal thermal injury can occur after radiofrequency (RF) ablation in the left atrium to treat atrial fibrillation. Existing methods to prevent esophageal injury have various limitations in deployment and uncertainty in efficacy. A new esophageal heat transfer device currently available for whole-body cooling or warming may offer an additional option to prevent esophageal injury. We sought to develop a mathematical model of this process to guide further studies and clinical investigations and compare results to real-world clinical data.

**Results:**

The model predicts that the esophageal cooling device, even with body-temperature water flow (37 °C) provides a reduction in esophageal thermal injury compared to the case of the non-protected esophagus, with a non-linear direct relationship between lesion depth and the cooling water temperature. Ablation power and cooling water temperature have a significant influence on the peak temperature and the esophageal lesion depth, but even at high RF power up to 50 W, over durations up to 20 s, the cooling device can reduce thermal impact on the esophagus. The model concurs with recent clinical data showing an 83% reduction in transmural thermal injury when using typical operating parameters.

**Conclusions:**

An esophageal cooling device appears effective for esophageal protection during atrial fibrillation, with model output supporting clinical data. Analysis of the impact of ablation power and heart wall dimensions suggests that cooling water temperature can be adjusted for specific ablation parameters to assure the desired myocardial tissue ablation while keeping the esophagus protected.

## Background

Esophageal injury is known to occur during ablation of the left atrium with radiofrequency (RF) energy, and the extreme outcome of this injury, atrio-esophageal fistula, may occur in up to 0.25% of patients [1, 2]. Cooling the esophagus during RF ablation has been investigated in a variety of fashions, including through the use of balloon devices and via direct instillation of liquid into the esophagus [3–10]. A recent meta-analysis found a 61% reduction in esophageal lesions using direct instillation of cold liquid [11].

A whole-body temperature management device that operates through the esophagus (EnsoETM, Attune Medical, Chicago, IL, USA) became commercially available in 2015, and is being investigated for its potential to counteract unintended esophageal heating during RF ablation of the left atrium [12]. Clinical data are now available from a small 6-patient study from a single site, [13] and a larger 120-patient clinical study, [14, 15] with longer term follow-up showing efficacy recently presented [16].

The device is available for a range of patient temperature management needs, and provides a high flow rate of water through a closed-circuit multi-channel 12-mm-diameter cylindrical silicone tube placed in the esophagus analogously to a standard orogastric tube (images and video available at https://www.attune-medical.com/). The device has a large heat transfer capacity and is currently used in cooling mode for the reduction of patient core body temperature from febrile or normothermic states, in warming mode for the prevention of inadvertent perioperative hypothermia, and in a feedback-control mode (automatically warming or cooling) for a variety of temperature management needs [17–20]. In order to further investigate the potential of this new approach, and quantify the possible efficacy, we sought to develop a mathematical model of this process and evaluate performance over a range of expected operating conditions while comparing output to recently available clinical data.

## Results

The sweep of the parameter values (RF power and cooling temperature) resulted in multiple simulations to study the influence of those parameters in the maximum or peak temperature, the lesion depth and the fraction of damage for up to 20 s of ablation. The esophageal lesion depth was defined as the distance from the pericardium (fat)–esophagus interface to the farthest point along the line perpendicular to the catheter tip crossing the ablated tissues, which results in a fraction of damage over 2%. The last point was obtained using the free software Python by linear interpolation of the corresponding data from Comsol®.

A line based on, and perpendicular to, the catheter tip across the involved tissues (myocardium, pericardium and esophagus) was defined as a data set for evaluation of fraction of damage and temperature for these studies.

### RF power application and the relationship to the temperature profile

**Fig. 1:**
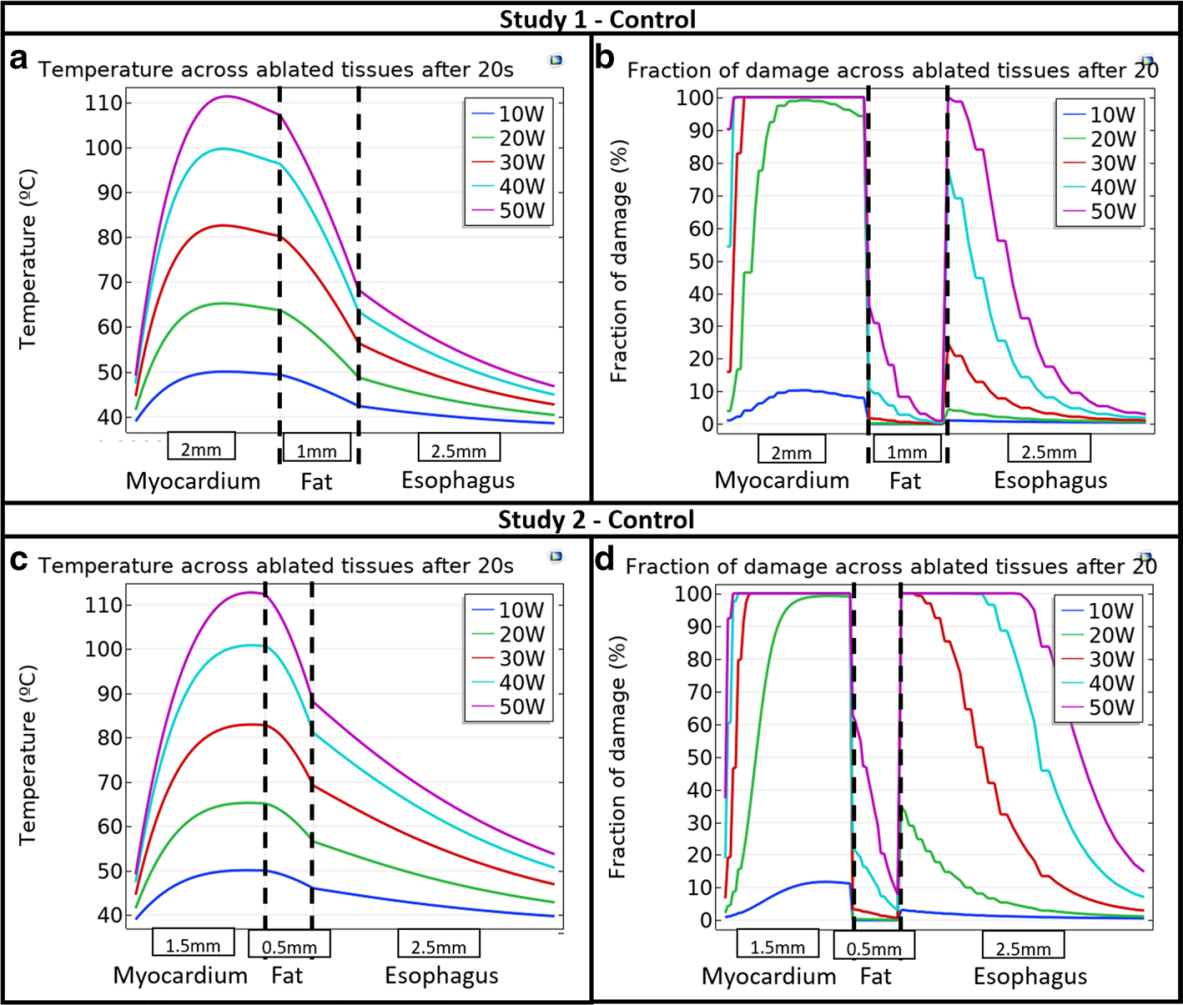
Temperature across ablated tissues for **a** Study 1 and **c** Study 2, and fraction of damage across ablated tissues for **b** Study 1 and **d** Study 2 after 20 s ablation time as a function of RF power applied

The temperature profiles were determined across the ablated tissues when power was varied from 10 to 50 W for the control situation (the cooling device not inserted) for both Study 1 and Study 2 during 20 s ablation duration. These profiles are shown in Fig. 1a, c, respectively. Similarly, the fraction of damage for the same situation in Study 1 and Study 2 is shown in Fig. 1b, d, respectively. From this, the dependency on maximum temperature, esophageal and myocardial lesion depth, and fraction of damage on RF power can be appreciated, showing the expected association (increasing peak temperature, fraction of damage and lesion depth as RF power is increased). Note that at higher wattages, typical durations of ablation are in the range of 8–10 s or less; therefore, peak temperatures shown here are not expected to occur. Data derived from values in Fig. 1 for ablation peak temperature and esophageal lesion depth and maximum fraction of damage as a function of RF power for Control 1 and Control 2 (control situation for Study 1 and Study 2) are shown in Table 1. The comparison between the results for Control 1 and Control 2 for each value of RF power applied is useful to quantitatively compare the implications of different anatomical dimensions in the lesion formation.

**Table 1:** Control peak temperature and lesion results as a function of RF power for Study 1 and Study 2

Power (W)	Study	Esophageal lesion depth (mm)	Maximum esophageal fraction of damage (%)	Peak temperature (°C)
10	Control 1	0.00	1.14	50.07
	Control 2	0.52	3.22	50.11
20	Control 1	0.77	4.56	65.25
	Control 2	1.88	34.71	65.29
30	Control 1	1.72	24.68	82.56
	Control 2	2.50	100.00	82.96
40	Control 1	2.44	77.44	99.63
	Control 2	2.50	100.00	100.85
50	Control 1	2.50	100.00	111.31
	Control 2	2.50	100.00	112.76

Numerical values are from Fig. 1

### Esophageal protection with the cooling device

**Fig. 2:**
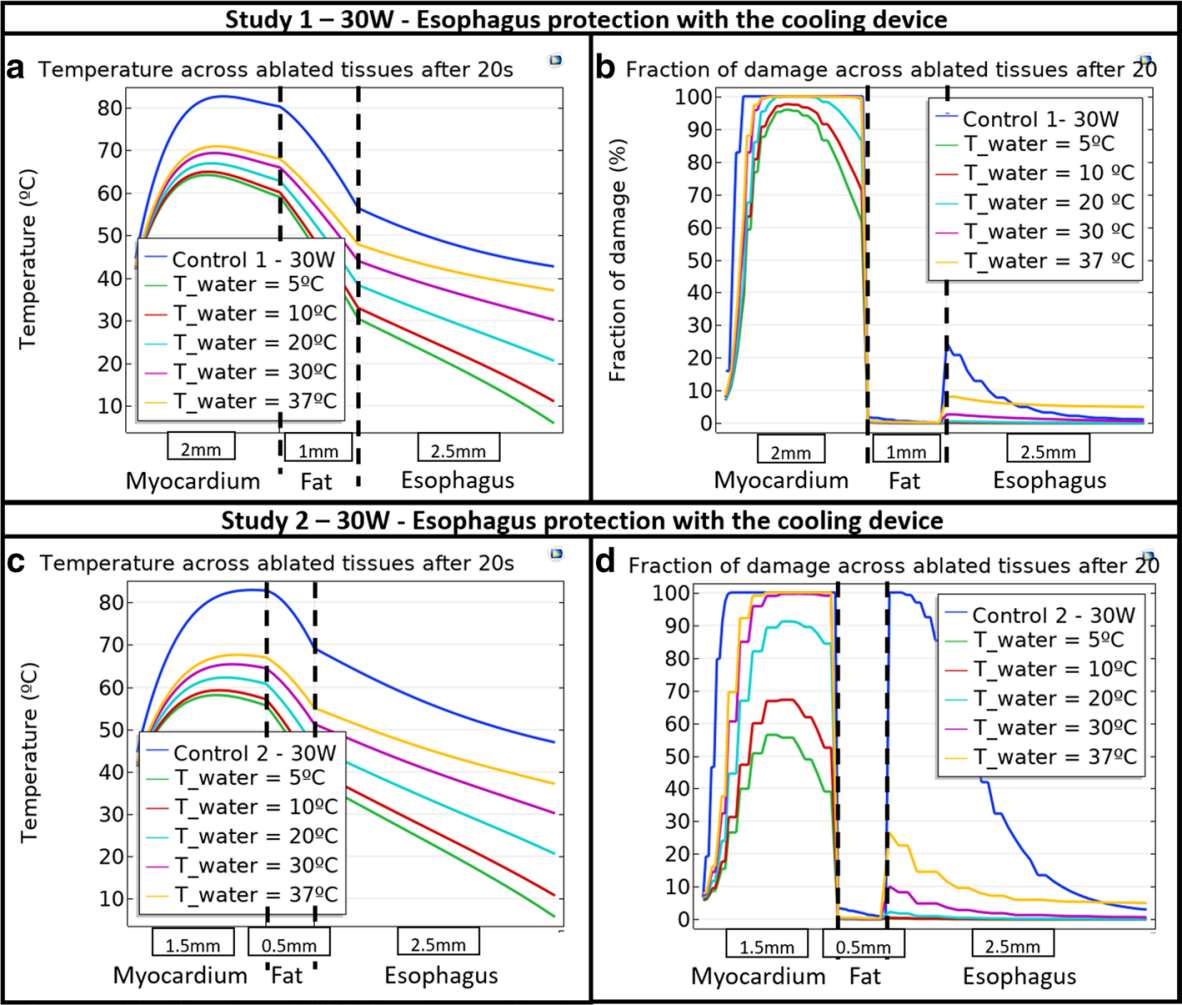
Temperature across ablated tissues for **a** Study 1 and **c** Study 2, and fraction of damage across ablated tissues for **b** Study 1 and **d** Study 2 after 20 s ablation time as a function of cooling water temperature and control at 30 W RF power

We next performed simulations with esophageal cooling in place. Figure 2 shows the comparison of control situation at 30 W RF power against the situation with esophageal protection from thermal injury achieved by the cooling device at different cooling water temperatures (T_water) and demonstrates how lesion formation and depth changes with cooling water temperature. The temperature across ablated tissues is shown in Fig. 2a, b for Study 1 and Study 2, respectively, while the fraction of damage is shown in Fig. 2c, d, respectively.

In a similar way to the control situation, the relevant quantitative data of peak temperature and esophageal lesion depth and maximum fraction of damage was obtained from Fig. 2, corresponding to the esophageal protection studies, and are presented in Table 2. The control values at 30 W RF power for both Study 1 and Study 2 are compared against different cooling device water temperatures (T_water) in Table 2.

**Table 2:** Cooling device protection peak temperature and lesion results as a function of RF power for Study 1 and Study 2 when RF power is 30 W

Study	Esophageal lesion depth (mm)	Maximum esophageal fraction of damage (%)	Peak temperature (°C)
Control Study 1	1.56	81.21	120.17
T_Water = 37 °C	2.50	14.87	67.79
T_Water = 30 °C	0.52	4.59	63.30
T_Water = 20 °C	0.00	0.61	56.29
T_Water = 10 °C	0.00	0.11	50.43
T_Water = 5 °C	0.00	0.04	48.33
Control Study 2	2.50	100.00	82.96
T_Water = 37 °C	2.50	29.62	67.88
T_Water = 30 °C	0.93	11.31	65.61
T_Water = 20 °C	0.00	2.57	62.46
T_Water = 10 °C	0.00	0.61	59.61
T_Water = 5 °C	0.00	0.33	58.44

Numerical values are from Fig. 2

**Fig. 3:**
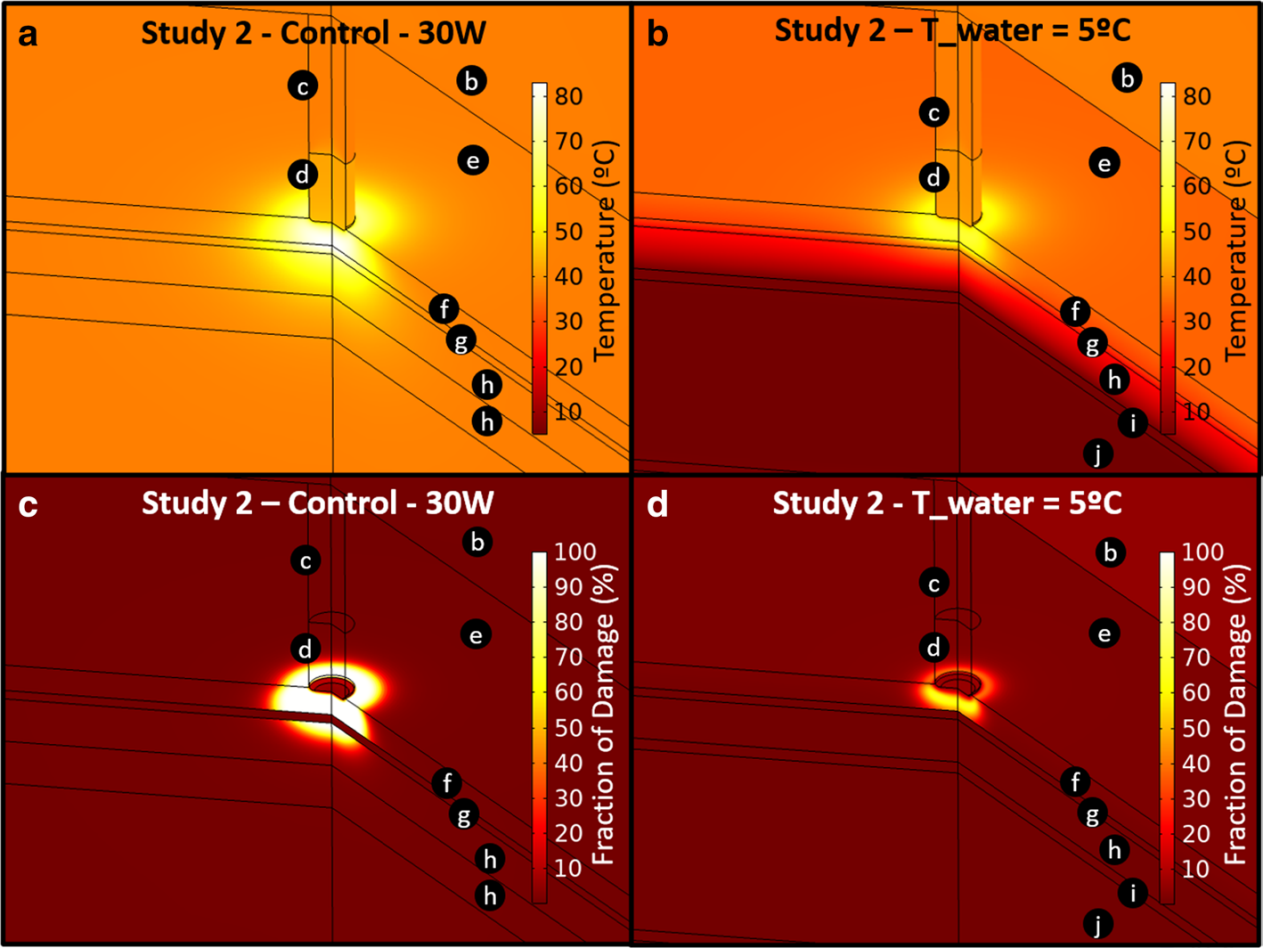
Temperature surface plot around ablated tissues for Study 2. **a** Control and **b** with the cooling device at 5 °C for 30 W RF power. Fraction of damage surface plot around ablated tissues for Study 2 **c** Control and **d** with the cooling device at 5 °C for 30 W RF power after 20 s ablation time. The subdomains are marked by letters in black circles: **c** catheter body; **d** catheter tip; **e** blood; **f** myocardium; **g** pericardium (fat layer); **h** esophagus; **i** cooling device wall; **j** cooling device water

The qualitative results for a 270 º 3D revolution of the original 2D axisymmetric geometry corresponding to Study 2 are presented in Fig. 3, which compares the control situation at 30 W and the esophageal protection when the cooling device water temperature (T_water) is 5 °C, the lowest value considered. Figure 3 shows the temperature distribution as well as the lesion formation shape in terms of the fraction of damage in the ablated tissues. The temperature surface plots for control at 30 W and the esophagus protection with T_water = 5 °C are shown in Fig. 3a, b, respectively, while the fraction of damage is presented in Fig. 3c, d, respectively. For the temperature and fraction of damage profiles in Fig. 3, the color range value was adjusted to be the same, so the qualitative comparison is clearer and more understandable. The black circles with letters in Fig. 3 denote the computational subdomains as specified in Fig. 6.

**Fig. 4:**
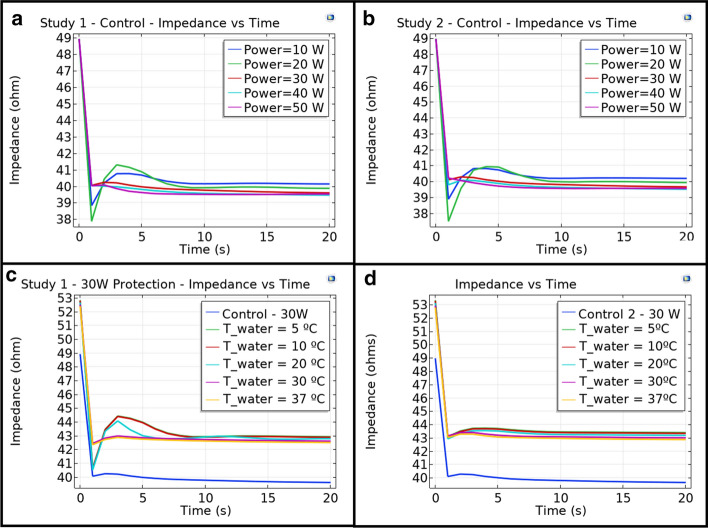
Control impedance vs. time plots for different RF power values for **a** Study 1 and **b** Study 2. Cooling device protection impedance vs. time plots for 30 W RF power and different values of cooling water temperature

A widely used parameter implied (and utilized as a control mechanism in the actual RF ablation procedures) is the impedance fall, and these values are available in the clinical trial data. Simulated data for impedance fall, measured as the magnitude of the relation between the terminal (catheter tip) voltage and current, were determined and are presented in Fig. 4. Figure 4a, b shows results for Study 1 and Study 2 control situation as a function of RF power applied. Figure 4c, d shows results for Study 1 and Study 2 using esophageal protection with the cooling device as a function of the cooling water temperature (T_water) when ablation power is 30 W. The control line is shown as well for further comparison.

**Fig. 5:**
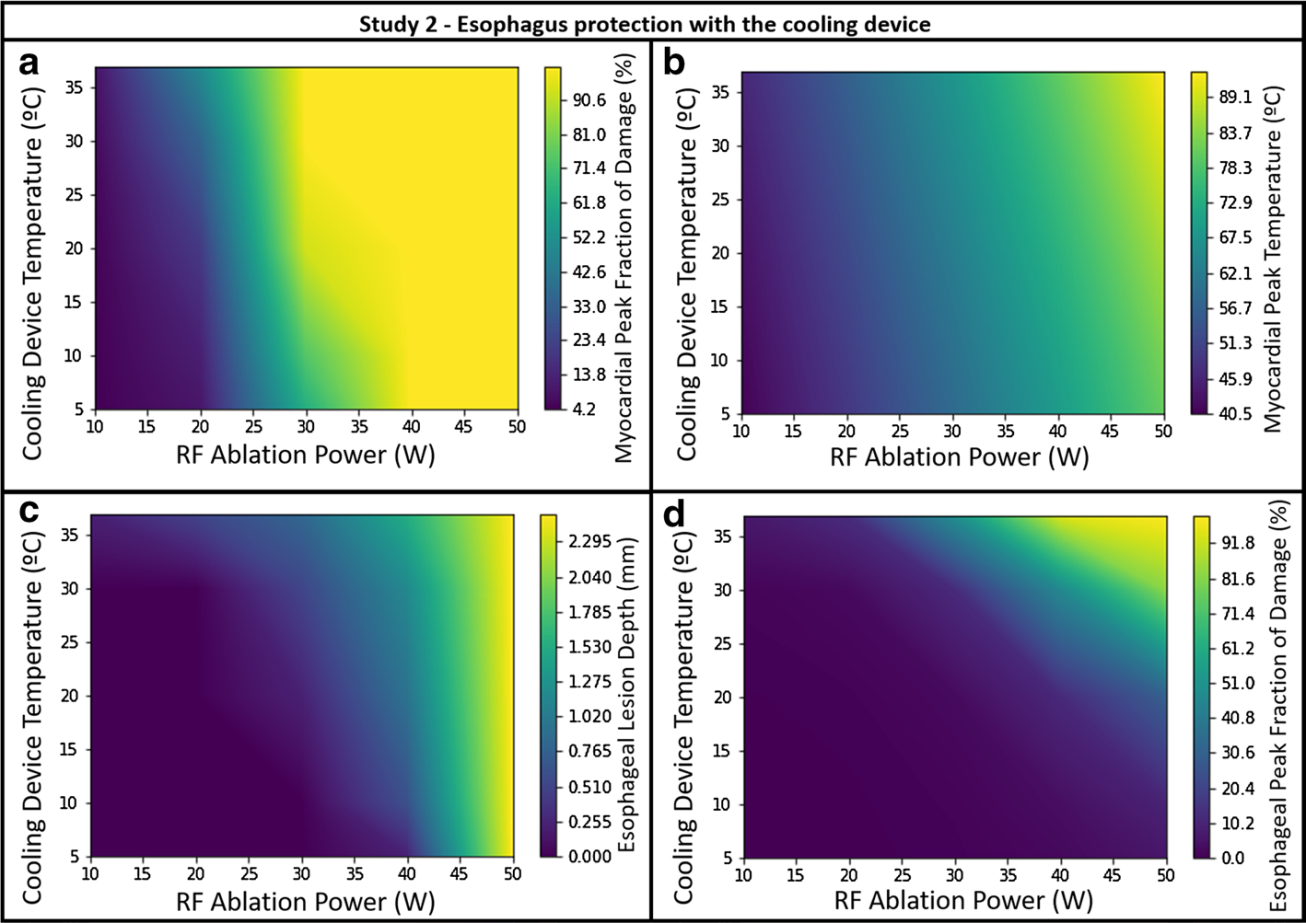
Contour plots for Study 2: **a** myocardial peak fraction of damage; **b** myocardial peak temperature; **c** esophageal lesion depth and **d** esophageal peak fraction of damage after 20 s ablation time as a function of cooling water temperature and RF power

Finally, we created contour plots for Study 2, to examine the influence of parameters simultaneously. Contour plots showing esophageal and myocardial peak fraction of damage (Fig. 5a, d, respectively), the myocardial peak temperature (Fig. 5b) and the esophageal lesion depth (Fig. 5c) and as a function of both RF power and cooling water temperature (T_water) are shown in Fig. 5. In these, with a given set of input operating parameters, an adjustment to cooling water temperature can be made such that optimal protection can be provided to the esophagus while keeping the desired myocardial damage.

## Comparison to clinical data

The results of a 120-patient clinical trial involving RF ablation procedure for the treatment of atrial fibrillation are presented in Tables 3 and 4. Patient and procedure characteristics are presented in Table 3, while the lesion grade of damage, classified from grade 1 to grade 6 is presented in Table 4.

**Table 3:** Patient and procedure characteristics in the clinical trial for both protected esophagus and control studies

Patient and procedure characteristics	Protected (*n* = 60)	Control (*n* = 60)
Male	*n* = 36 (60%)	*n* = 37 (61.7%)
Age (years)	65 ± 10	65 ± 9
LA diameter—anteroposterior (cm)	4.10 ± 0.9	4.20 ± 0.6
LV ejection fraction (Simpson’s) (%)	55 ± 9	52 ± 8
BMI (kg/m^2^) (%)	28.5 ± 5.3	29.80 ± 7
Persistent, 1st time ablation	*n* = 27 (45%)	*n* = 30 (50%)
PAF, 1st time ablation	*n* = 24 (40%)	*n* = 20 (33.40%)
Repeat left atrial ablation	*n* = 8 (13.3%)	*n* = 9 (15%)
LAT (left atrial tachycardia)	*n* = 1 (1.7%)	*n* = 1 (1.7%)

**Table 4:** Lesion grade results for the clinical trial

Endoscopy results (*n* = 120)	Protected (*n* = 60)	Control (*n* = 60)
No lesion	56	42
Grade 1 (erythema)	0	4
Grade 2 (erosion < 5 mm)	1	1
Grade 3 (erosion(s) > 5 mm)	0	1
Grade 4a (superficial ulcer, clean)	1	4
Grade 4b (superficial ulcer with clot)	0	1
Grade 5a (deep ulcer, clean)	0	1
Grade 5b (deep ulcer with clot)	0	0
Grade 6 (fistula)	0	0

Because results show only endoscopic outcome (and not tissue depth of injury, which would require a separate surgical procedure or autopsy to determine), we rely on the reduction in esophageal lesion formation of 83% as a marker of efficacy. When compared with model output, using similar operating parameters, and considering the expected variation in tissue thickness across patients and within the region of interest in each patient, the findings appear in line with predictions.

## Discussion

We show here for the first time the protective effect of a new esophageal cooling device from RF energy applied to the atrium and evaluate this effect across a range of operating parameters that encapsulate the expected values in clinical use. These findings suggest that the protective effects of this approach may be significant, and moreover, that these effects can be adjusted, or tuned, by adjusting coolant temperature, such that optimization of esophageal protection can be pursued while minimizing or eliminating any reduction of ablation efficiency in the atrial wall. Comparison of model predictions with actual clinical data further reinforces the value of this model.

Several variables impact the efficacy of this approach, with the temperature of the coolant (water) having perhaps the greatest impact in the lesion depth and peak temperature, as might be expected. Also, the anatomical dimensions of the patient have a significant effect in the results as evidenced by the difference in the results for Study 1 and Study 2. Peak temperature and the esophageal lesion depth and fraction of damage are considerably influenced by the RF power. Figure 1a, c shows the temperature profile across the ablated tissues as a function of RF power. It is evident that the temperature begins rising from the catheter tip until reaching a peak temperature and then falls with different slopes, depending on the dimensions considered. Apparently, the peak temperature varies almost linearly with RF power. The fraction or percentage of damage shown in Fig. 1b, d shows a non-regular shape, which can be attributed to the nature of the fraction of damage calculation (Eqs. 8, 9), and to the different material properties (frequency factor and activation energy) considered for the myocardial and the esophagus compared with those set up for the fat layer [21]. Additionally, Fig. 1b, d suggests 10 W is the only power applied leading to weak myocardial damage for both anatomical dimensions studied in Study 1 and Study 2, in turn suggesting that for low wattage, a longer duration is generally required to obtain adequate lesion formation. For RF powers from 20 to 50 W, the myocardial lesion reaches 100% depth in almost all regions, but the pericardial (fat layer) lesion is considerably higher as the power is increased. Nevertheless, it also increases the undesired esophageal damage, especially for the case of very thin cardiac walls considered in Study 2. These extreme anatomical conditions lead to both larger esophageal lesion depth and percentage of damage, suggesting a thin cardiac wall could lead to greater probability of the lethal atrio-esophageal fistula.

The results shown in Table 1 quantitatively support what is graphically evident in Fig. 1, specifically that the maximum esophageal percentage of damage is considerably higher in Study 2 than in Study 1, highlighting that the degree of damage is greater with thin heart walls. As shown in Fig. 2 and Table 2, the esophageal protection against damage using the cooling device even with body-temperature water flow (37 °C) is seen in the reduction in some of the critical parameters considered in this study: the fraction of myocardial and esophageal damage, and the global peak temperature; but leads to a higher esophageal lesion depth, suggesting that 30 ºC cooling or lower is needed to have beneficial effect. The discontinuous steps observed in Fig. 2 are a result of changes in tissue properties, which differ for each tissue. The most influencing properties on the discontinuous steps are those related to the thermal damage measured by the Arrhenius equations (Eqs. 8 and 9).

As expected, the protective effect is more evident in Study 1 than in Study 2. This can be expected by the greater lesion depth predicted for the thin cardiac wall conditions in Fig. 1. Nevertheless, the cooling device considerably reduces esophageal damage for both cardiac dimensions considered. On the other hand, despite the fact that pericardial (fat) damage is also reduced with the placement of the cooling device and with the reduction of the cooling water flowing inside it, the myocardial lesion remains almost unchanged when the water temperature is around 30 ºC (which is the aim of RF ablation therapies for the treatment of atrial fibrillation). A reduction of the desired cardiac injury is possible at lower water temperatures in some anatomic conditions; however, recently presented clinical data showing longer term follow-up has shown equivalent efficacy of the ablation procedure with and without the cooling device, despite a marked reduction in esophageal lesion formation [16]. Use of the esophageal device appears to impact temperature and tissue damage more significantly at the esophagus than at the myocardium. Moreover, adjustment of temperature can be performed to optimize protection while minimizing any impact on myocardial lesions, if needed. Figure 3 further shows that esophageal cooling provides a notable reduction in esophageal lesion depth compared to the case of the non-protected esophagus.

The results in Fig. 3 for Study 2 suggest a regular shape for the temperature distribution, and as expected, it is evident that the cooling water in the device acts as a thermal barrier for the esophageal protection. In contrast, the shape of the lesion formation in terms of the fraction of damage is irregular, suggesting the greater influence of the parameter is the Arrhenius expression for the measurement of tissue thermal damage (Eqs. 8 and 9). Although myocardial damage can also be affected by the cooling water temperature (for example, the fraction of damage and the size of the injury may both be reduced when very low temperatures as 5 °C are used), as mentioned above, recent clinical data suggest that any impact is clinically insignificant [16].

The impedance falls for the control situation in both Study 1 and Study 2 (Fig. 4a, d) suggest that increasing the power slightly increases the final impedance fall. Nevertheless, the values are very similar within different powers and anatomical dimensions. For the esophageal protection, it is evident from Fig. 4c, d that the placement of the cooling device increases the initial impedance when compared to control, but the impedance fall remains similar, without significant influence from the cooling water temperature. The clinical data show an average 122 Ω impedance and a drop of 9 + − 5 Ω, suggesting the simulation results are considerably close to those reported clinically.

We included a wide range of energy deposition in our model, including a high range that is generally beyond what most practitioners aim to achieve; however, since the trend in current ablation practice is towards higher energy, but shorter duration, inclusion of a wider range of energy in this model is appropriate. Moreover, even when not intending to deploy higher energy, it is often the case that sufficient energy is deployed to result in a steam pop. Steam pops occur when tissue temperature exceeds 100 °C, and in fact, experimental measurements have found tissue temperatures of 102 ± 17 °C reached during steam pop formation [22]. Steam pops are common when cooled electrode temperature exceeds 40 °C and are not predictable from power or impedance drop, but small impedance rises and sudden drops in measured electrode temperature indicate possible steam formation [23]. Investigators have found that the incidence of steam pops significantly increased for both nonirrigated and irrigated ablations at 40 W [24] and have noted that the disparity between catheter and tissue temperatures during irrigated RF ablation frustrates one's ability to predict steam pops [25].

The contour plots shown in Fig. 5 offer a means to determine which parameter (ablation power or cooling device temperature) should be adjusted to minimize esophageal damage while maximizing the desired cardiac injury. Figure 5 highlights the esophageal lesion depth and both myocardial and esophageal maximum fraction of damage and the myocardial peak temperature as a function of cooling water temperature and the RF power applied, which allows selection of appropriate cooling water temperature in order to protect the esophagus from thermal damage while assuring the desired myocardial tissue ablation. Pairs of values for different parameters can be taken from these graphs to assure both low esophageal lesion depth and maximum fraction of damage and high myocardial peak fraction of damage.

In summary, a 2D axisymmetric model of an esophageal heat transfer device currently available for whole-body cooling or warming shows significant protective effects on the esophagus against thermal damage from RF energy ablation. The model supports growing clinical data now available.

## Limitations

Although mathematical modeling offers valuable insight into physical phenomena such as those investigated in this study, and these results serve as a guide to expected clinical results, some variation in the results found in clinical application should be expected. The results are expected to vary with parameters outside of those considered in this study. For example, tissue properties may vary between patients, as may the contact force and ablation power. Nevertheless, all these parameters can easily be studied in silico with the model proposed here. Future work should pave the way to build computational models aimed to simulate the RF ablation based on the ablation index instead of fixed values for the various parameters in order to have improved reference cases to compare model predictions with clinical data and to increase reliability. We modeled a wide range of energy depositions during ablation, and the high end of this range of energy deposition is actually beyond what is typically done in real practice. As such, extremes of temperature are shown in our results that occur at higher ranges of energy deposition; however, as noted in the Discussion, the occurrence of steam pops resulting from high energy deposition is likely more frequent than appreciated, and with trends towards higher energy usage in current clinical practice, these higher temperatures are important to highlight.

## Conclusions

An esophageal cooling device appears effective for esophageal protection during atrial fibrillation, with model output supporting clinical data now available. Analysis of the impact of RF power, ablation time, cooling water temperature and tissue thermal conductivity suggests that cooling water temperature can be adjusted for specific ablation parameters to assure the desired cardiac tissue ablation while keeping the esophagus protected.

## Methods

Comsol Multiphysics® was used to model and simulate the process of RF ablation of the left atrium for two situations. The first (Study 1) contemplates a collapsed esophagus in contact with the left atrium, while the second (Study 2) includes the presence of the esophageal cooling device being investigated, circulating a range of water flow temperatures.

### Computational domain

**Fig. 6:**
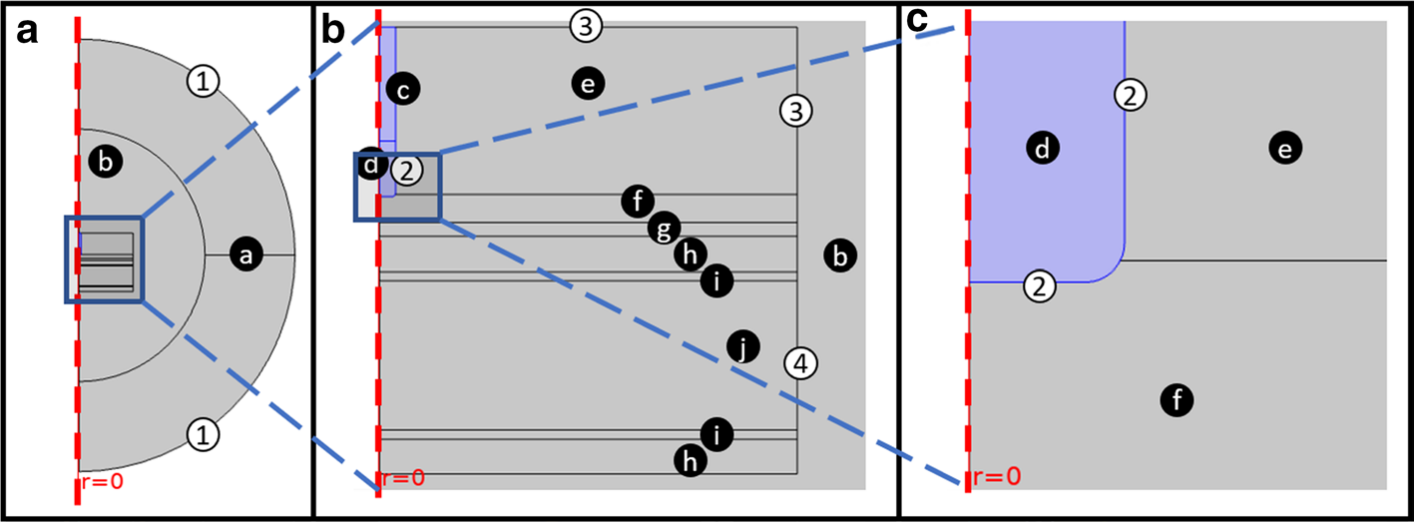
Computational domain (geometry): **a** general view, **b** zoom-in of the ablation area, and **c** zoom-in of the catheter tip. The subdomains are marked by letters in black circles: **a** thoracic cavity (infinite elements); **b** thoracic cavity; **c** catheter body; **d** catheter tip; **e** blood; **f** myocardium; **g** pericardium (fat layer); **h** esophagus; **i** cooling device wall; **j** cooling device water. The relevant boundary conditions are marked by numbers in white circles: (1) external boundaries, (2) catheter tip boundaries, (3) left atrium blood inlets, (4) cooling device water inlet

Figure 6 shows the computational domain composed by a simplified 2D axisymmetric model of the left atrium in contact with the esophagus protected by the cooling device and immersed in the thoracic cavity. A surrounding infinite elements domain is defined in Comsol to truncate the model and consider an infinitely extended domain without increasing the computational size and cost. The ablation catheter is inserted in the blood subdomain and is in contact with the inner wall of the myocardium. The cooling device subdomain is not considered in the computations for the collapsed esophagus model (control).

The thickness of myocardium, pericardium (fat layer) and esophagus walls were set at two combinations (Study 1 and Study 2) as specified in Table 5. [26, 27] The dimensions were chosen to consider two different left atrium posterior wall anatomies and analyze how much the esophageal lesion depth results are affected by different tissue thicknesses. The catheter tip radius was set to 1.1665 mm and the insertion depth was set to 160 µm, corresponding to 1/25 of the tip height (4 mm). As an infinite element domain was used to guarantee a large enough domain, the non-mentioned dimensions are not critical.

**Table 5:** Left atrium posterior wall anatomy dimensions considered in Study 1 and Study 2

Study	Myocardium thickness (mm)	Fat thickness (mm)	Esophagus thickness (mm)
1	2.00	1.00	2.50
2	1.50	0.50	2.50

### Governing equations and boundary conditions

Maxwell equations govern electromagnetism. The ACDC module from Comsol® was used to model electromagnetic phenomena at low frequency. Because the magnetic skin depth in human tissues is known to be very large when compared with tissue dimensions, only electric currents were considered as a heating source. The *Electric Currents* interface from the ACDC module was used to solve the equations for current conservation (Eqs. 1–4). The energy balance governs heat transfer. The Pennes’ approximation allows us to obtain the bioheat transfer equation, which accounts for heat sources from blood perfusion and metabolism in the classical heat transfer equation. The associated Eq. 5 is solved through the *Bioheat Transfer* interface from the Heat Transfer module from Comsol®. The term Q represents the external heat source, which in this case is the electromagnetic, volumetric and surface losses due to applied RF power, and given by the expression in Eq. 6. The term Q_bio_ in Eq. 7 corresponds to the sum of the heat produced by blood perfusion and the metabolic heat source.

1 $$ \nabla \cdot {\mathbf{J}} = {\text{Q,}} $$

2 $$ {\varvec{J}} = \sigma {\varvec{E}} + j\omega {\varvec{D}} + {\varvec{J}}_{{\varvec{e}}} , $$

3 $$ {\varvec{E}} = - \nabla {\text{V,}} $$

4 $$ {\varvec{D}} = \varepsilon_{0} \varepsilon_{r} {\mathbf{E}}, $$

5 $$ \rho c_{p} \frac{\partial T}{{\partial t}} + \rho c_{p} {\varvec{u}} \cdot \nabla {\text{T}} + \nabla \cdot \left( { - {\text{k}}\nabla {\text{T}}} \right) = {\text{Q}} + {\text{Q}}_{bio} , $$

6 $$ Q = \frac{1}{2}Re\left( {{\varvec{J}} \cdot {\varvec{E}}^{\user2{*}} } \right), $$

7 $$ Q_{{\text{bio}}} = \rho_{b} C_{p,b} \omega_{b} \left( {T_{b} - T} \right) + Q_{met} . $$

For electric governing equations, **J** is the current density, *Q* is the current source term, *σ* is the electric conductivity, **E** is the electric field intensity, **D** is the electric displacement, ⍵ is the angular frequency, **Je** is the external current density and *V* is the electric potential. For thermal governing equations: *T* is the temperature, *⍴* is the density, *C*_p_ is the heat capacity, **u** is the fluid velocity, *k* is the thermal conductivity, *Q* is the heat source term (which corresponds to resistive heating in this case). The heat due to blood perfusion is given in terms of blood perfusion rate ω_b_, the subscript *b* indicates blood (the metabolic heating source *Q*_met_ was neglected). To account for convective transport while avoiding the complexity of a full fluid dynamics study, the blood domain was replaced by a convective heat flux boundary condition. The heat transfer coefficient between blood and tissue was *h*_t_ = 610 W/(m^2^K), and between blood and ablation catheter *h*_e_ = 3346 W/(m^2^K) [28]. This way, the model considers an average situation for the flow of blood. On the other hand, to couple RF energy and bioheat transfer, the multiphysics interface *Electromagnetic Heat Source* from the Multiphysics module of Comsol was used to account for electromagnetic volumetric and surface losses.

The governing equations (1–7) are for the whole computational domain, and changes within domains are based on the material properties related to those equations. The bioheat terms in Eq. 5 only apply for tissue domains: myocardium, esophagus, fat layer and thoracic cavity (domains a, b, f, g, and h). The electromagnetic heat source was applied to all domains. Besides the material properties, boundary and initial conditions are also needed to solve this set of partial differential equations. Figure 6 shows the relevant boundaries in the model, while Table 6 describes the associated thermal and electrical boundary conditions. The electrical boundary conditions for the catheter tip considered power values from 10 to 50 W by means of the “Terminal” boundary condition feature, which is available in the Electric Currents interface of Comsol®. The cooling water temperature inlet was set from 5 to 37 °C. The initial temperature was 37 °C (body temperature) while the initial electric potential was set to 0 V.

**Table 6:** Thermal and electrical boundaries conditions

Boundary	Description	Electrical boundary condition	Thermal boundary condition
1	External boundaries	Ground	Thermal Isolation
2	Catheter tip boundaries	Power = (10, 20, 30, 40, 50) W	N/A
3	Left atrium blood inlets	N/A	T = 37 °C
4	Cooling device water inlet	N/A	T = (5, 10, 15, 20, 30, 37) °C

### Thermal damage analysis

To evaluate the fraction of damage in the ablated tissues (domains a, b, f, g, and h), a thermal damage analysis was performed based on the Arrhenius equation (Eq. 8). [21, 29–31] Where α is the degree of tissue injury, A is the frequency factor, ΔE is the activation energy, R is the gas constant and T is the temperature. For myocardium, esophagus and average tissue domains (a, b, f, and h), A = 2.94e38[1/s], ΔE = 2.596e5[J/mol]. For the pericardial fat domain (g), A = 4.43e16[1/s], ΔE = 1.3e5[J/mol]. The fraction of damage is evaluated with the expression in Eq. 9 and is valued between 0 and 1, or between 0 and 100, which represents the fraction of damage as a percentage of tissue thickness.

8 $$ \frac{d\alpha }{{d{\text{t}}}} = \left( {1 - \alpha } \right)Ae^{{ - \frac{\Delta E}{{RT}}}} $$

9 $$ \theta_{d} = {\min}\left( {\max \left( {\alpha ,0} \right),1} \right) $$

### Tissue and material properties

The electrical and thermal tissue and material properties are presented in Table 7. To account for the dependence of tissue material properties with the temperature, interpolation functions were defined for electrical conductivity σ, thermal conductivity k and density ρ in Eqs. 10 to 12, respectively. Some of the related functions are available in the Comsol material library [32, 33]. For the water and blood domains, predefined Comsol functions for liquid water available in the material library were used. The mean values for the predefined functions are shown in Table 7.

11 $$ \begin{gathered} \sigma_{a} \left( {T\left[ K \right]} \right) = \left\{ {\begin{array}{ll} {\left( {9e - 3\left( {T - 313.15} \right) + 0.42} \right) \quad {\text{if}} 293.15 \le T < 348.15} \\ {\left( {9e - 3\left( {348.15 - 313.15} \right) + 0.42} \right) \quad {\text{if}} 348.15 \le T < 358.15} \\ {\begin{array}{ll} {\left( { - 3.5e - 3\left( {T - 358.15} \right) + 0.735} \right) \quad {\text{if}} 358.15 \le T < 378.15} \\ {\left( { - 52e - 3\left( {T - 378.15} \right) + 0.57} \right) \quad {\text{if}} 378.15 \le T < 388.15} \\ \end{array} } \\ \end{array} } \right., \hfill \\ k_{a} \left( {T\left[ K \right]} \right) = 0.493\left( {1 + 1.2e - 3\left( {T - 310.15} \right)} \right) \quad {\text{if}} 276.15 \le T < 373.15 , \hfill \\ \end{gathered} $$

12 $$ \rho_{a} \left( {T\left[ K \right]} \right) = 0.493\frac{{1 + 1.2e - 3\left( {T - 310.15} \right)}}{{\left( {3212} \right)\left( {1.474e - 7\left( {1 + 3.39e - 3\left( {T - 310.15} \right)} \right)} \right)}} \quad {\text{if}} 276.15 \le T < 373.15. $$

**Table 7:** Tissue and material properties

Subdomain	Electrical properties		Thermal properties		
	*σ* (S/m)	ε	*k* (W/(m K))	*C*_p_ (J/(kg K))	*ρ* (kg/m^3^)
Myocardium, esophagus, and thoracic cavity	*σ*_a_(T)	8000	*k*_a_(T)	3212	*ρ*_a_(T)
Silicone catheter body and cooling device	10e−12	11.7	130	700	2329
Platinum catheter tip	8.9e6	1	71.6	133	21,450
Blood	1.50	2.2e4	N/A	N/A	N/A
Pericardium fat layer	0.5	80	0.21	2348	911
Cooling device water	5.5e−6	75	~ 0.5	~ 4000	~ 1000

### Meshing

**Fig. 7:**
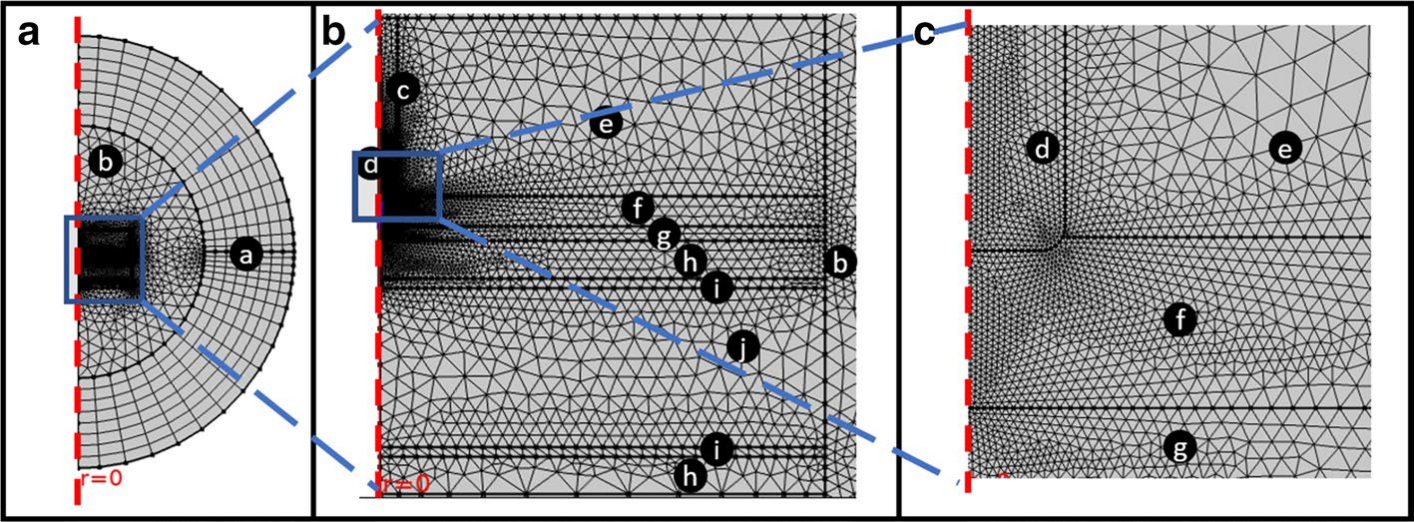
Free triangular mesh refined and around the catheter tip region. The infinite elements domain was meshed using a mapped mesh. The complete mesh consists of 5748 for the collapsed esophagus and 6234 with the cooling device inserted

The mesh utilized the meshing option “physics controlled” with a predefined tetrahedral fine mesh size and a mesh refinement made in the area around the catheter tip, where we expect to find the most relevant changes in temperature. The mesh consisted of triangular elements: 5748 for the collapsed esophagus and 6234 with the cooling device inserted. Figure 7 presents the mesh with the same region zooming used in Fig. 6.

### Study type and solver

The *Frequency-Transient* study type from Comsol was used. This selection was made because the multiphysics phenomena that were analyzed involve alternating electric currents, which are convenient to solve with a frequency domain study, while the additional heat transfer phenomena are solved in the transient domain in order to appreciate the temperature profile changes over time. The frequency considered for the ablation RF energy was 500 kHz, and simulations were obtained from 0 to 20 s with a step of 1 s. For the case with esophagus protection with the cooling device, a precooling time of 5 min was applied before beginning the ablation. A parametric sweep was performed to simulate all the combinations of specified values for ablation power and cooling water temperature (Table 6).

### Comparison with clinical data

**Fig. 8:**
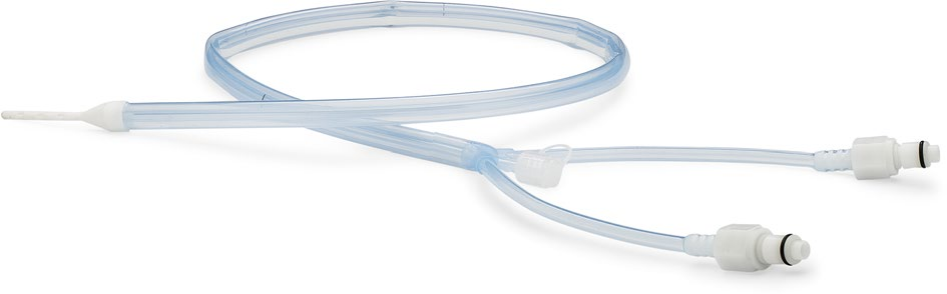
Intraesophageal cooling device EnsoETM®

Recently presented clinical data from a randomized controlled trial enrolling 120 patients are available and were used for comparison to model predictions [14–16]. Ablation settings were 30 W posteriorly, 40 W anteriorly with Ablation Index targets of 350–400 posterior, 450–500 anterior. Because use of the Ablation Index (calculated from time, contact force and power) eliminates a firm time cut-off, and because the coefficients of the Ablation Index are proprietary, we extrapolated ablation times from procedures performed prior to use of the Ablation Index, where if contact force is high, the delivery is just over 8 s, with a 20 s upper-limit cut-off otherwise. The clinical data measuring esophageal protection with the cooling device as well as the control conditions were compared with the simulated results to validate the mathematical model reliability and prediction power. The esophageal cooling device is shown in Fig. 8; reprinted with permission from Attune Medical.

## Data Availability

The datasets used and/or analyzed during the current study are available from the corresponding author on reasonable request.
